# Engagement With a Smartphone-Delivered Dietary Education Intervention and Its Relation to Dietary Intake and Cardiometabolic Risk Markers in People With Type 2 Diabetes: Secondary Analysis of a Randomized Controlled Trial

**DOI:** 10.2196/71408

**Published:** 2025-05-30

**Authors:** Linnea Sjoblom, Freja Stenbeck, Ylva Trolle Lagerros, Essi Hantikainen, Stephanie E Bonn

**Affiliations:** 1 Division of Clinical Epidemiology, Department of Medicine, Solna, Karolinska Institutet, T2, Maria Aspmans gata 16, Stockholm, 171 76, Sweden, 46 8-517 791 83; 2 Department of Medicine, Huddinge, Karolinska Institutet, Stockholm, Sweden; 3 Institute for Biomedicine, Eurac Research, Bolzano, Italy

**Keywords:** adherence, dietary change, diabetes mellitus, type 2 diabetes mellitus, healthy diet, mHealth, smartphone app, user engagement, mobile phone

## Abstract

**Background:**

Mobile health (mHealth) interventions offer a promising way to support healthy lifestyle habits, but effectiveness depends on user engagement. Maintaining high user engagement in app-based interventions is important, yet challenging.

**Objective:**

We aimed to examine the association between user engagement with an app-based dietary education for people with type 2 diabetes and changes in diet quality, dietary intake, and clinical measures.

**Methods:**

In this randomized clinical trial, people with type 2 diabetes were recruited within primary care and randomized 1:1 to a 12-week smartphone-delivered app-based dietary education or control group. Participants were followed up after 3, 6, and 12 months. Dietary intake was assessed using a food frequency questionnaire. The control group received the app at the 3-month follow-up. User engagement was analyzed among all participants. Categories of high (100%), moderate (50%‐99.9%), and low (<50%) user engagement were created based on the percentage of activities completed in the app. We used paired *t* tests to compare mean changes in diet quality, dietary intake, and clinical markers within user engagement groups, and fitted linear regression models to analyze differences in change between groups.

**Results:**

Data from 119 participants (60.5%, 72/119 men) were analyzed. The mean age at baseline was 63.2 (SD 10.3) years and mean BMI was 30.1 (SD 5.1) kg/m^2^. User engagement was high with an average of 77.1% of app activities completed. More than half (53.8%, 64/119) of the users showed high user engagement, 21.8% (26/119) moderate, and 24.4% (29/119) low. Directly following the app-based education, a significant difference in change was seen for whole grains (β=20.4, 95%CI 0.57‐40.3) in participants with high user engagement compared to the low user engagement group who decreased their intake (*P*=.03). At follow-up after 6 to 9 months after completed education, significant differences in change were seen for fiber, wholegrains, carbohydrates, saturated fat, sodium, and total energy in the moderate compared with the low engagement group, and a significant difference in change was seen for carbohydrates in the high, compared with the low, user engagement group.

**Conclusions:**

User engagement was generally high for the smartphone-based dietary education, suggesting that an app targeting dietary habits is feasible to use. Those with higher user engagement seem to maintain healthier dietary behaviours over time, compared to those with low user engagement. Future mHealth interventions should focus on ways to engage those with low interest.

## Introduction

Lifestyle interventions, including dietary counseling, are commonly prescribed to people with type 2 diabetes in primary health care, often in combination with medications [1,2]. In recent years, the emergence of new mobile health (mHealth) apps has been proposed as a cost-effective means of delivering nutritional therapy [2-4]. mHealth interventions offer a promising way to support lifestyle changes, but the effect of any intervention is dependent on adherence, ie, the extent to which participants follow an intervention as intended [5-7]. Maintaining user engagement in an app-based intervention is challenging [8,9], yet, important.

Specific intervention features, such as personal tailoring, feedback, and reminders, are associated with higher engagement in digital interventions targeting chronic diseases [10,11]. In a smartphone-delivered intervention targeting healthy lifestyle behaviours, high app user engagement was associated with improvements in dietary quality and reductions in BMI and waist circumference at 1-year follow-up among individuals at increased risk for type 2 diabetes [12]. Furthermore, high user engagement with an app specifically targeting vegetable consumption was also associated with improved vegetable intake among Australian adults [13].

We have previously shown that a smartphone-delivered dietary education had positive effects on dietary fat intake and serum triglyceride levels in persons with type 2 diabetes [14]. In this study, we aimed to examine the association between user engagement with the app-based dietary education and changes in diet quality, dietary intake and clinical variables of BMI, waist circumference, body fat percentage, glycated haemoglobin (HbA_1c_), serum lipid levels, and blood pressure, directly after completing the education. We also aimed to study the association between user engagement and long-term change in dietary quality and dietary intake 6 to 9 months after the completed education.

## Methods

### Study Design

We have analyzed data from the HAPPY (Healthy eating using APP technologY) trial, previously described in detail [15]. The main results from the trial have been published [14]. In brief, this was a 2-armed randomized controlled trial comprising women and men with type 2 diabetes that were continuously recruited within 5 primary health care centrs in Stockholm, Sweden. Initial information about the study was provided by health care personnel at their routine health care visit. Patients interested in participating were contacted by study personnel to receive more detailed information. Those who agreed to participate were scheduled for a physical baseline meeting with study personnel. Participants were randomized 1:1 to a 12-week app-based dietary education intervention in addition to regular care, or a control group receiving regular care only. The primary aim of the trial was to examine the effectiveness of the intervention on dietary intake and cardiometabolic risk markers. Study inclusion criteria were: having type 2 diabetes, ≥18 years of age, being able to read and understand Swedish, and having access to and being able to use a smartphone with a personal e-identification. No exclusion criteria were applied. The data collection began in January 2019 and was finalized in August 2023. It was temporary paused during 2020 due to the COVID-19 pandemic. This study is reported according to the CONSORT-EHEALTH (Consolidated Standards of Reporting Trials of Electronic and Mobile HEalth Applications and onLine TeleHealth) checklist, which is developed for eHealth or mHealth interventions (Checklist 1).

### Ethical Considerations

The trial was conducted in accordance with the Declaration of Helsinki, and approved by the Ethics Committee of the Regional Ethical Review Board, Stockholm, Sweden (2018/652-31; 2018/1094‐32; 2018/2393‐32; 2020‐00591; 2020‐07005; and 2022-02557-02). It was also registered at ClinicalTrials.gov (NCT03784612). All study participants received oral and written information about the study before giving their written consent before the study started. The original informed consent allows secondary analysis of data without additional consent. Participants received no compensation for participation in the study and all data were anonymized after data collection.

The intervention group received the app at study start and the control group received the app at the 3-month follow-up. All participants responded to a web-based lifestyle questionnaire at baseline and again after 3 months (directly after completing the dietary education in the intervention group), 6 months (directly after completing the dietary education in the control group), and after 12-months of follow-up. The baseline questionnaire was filled out before participants were informed about their group allocation. We also measured weight (kg), height (cm), waist circumference (cm), body composition, blood pressure, serum levels of glycated haemoglobin (HbA_1c_), and lipids at baseline, and after 3- and 6 months of follow-up.

### Participant Flow and Outcome Assessments

In total, 133 individuals agreed to participate in the trial. Of these, 68 were randomized to the intervention and 65 to the control group. One participant randomized to the intervention group and 3 participants randomized to the control group dropped out before study start, ie, before knowing their group allocation and before providing any baseline data. In total 119 participants, 65 in the intervention group and 54 in the control group, downloaded the app.

In this study, we addressed change in diet quality, dietary intake, and clinical outcomes from baseline to directly after having completed the dietary education, hereafter written as “short-term” follow-up. Furthermore, we investigated the association on diet quality and dietary intake from baseline to 9 months after completing the dietary education for the intervention group and 6 months after completion in the control group, from here on written as “long-term” follow-up.

### The HAPPY Trial Intervention and App Content

The smartphone delivered dietary education focused on the overall diet in accordance with current evidence-based guidelines and the Swedish national dietary recommendations [16]. The app design included the health belief model [17], stages of change model [18], and social cognitive theory [19]. We further included behavior changes techniques such as educational information, goal setting, self-monitoring, feedback, and performance [20]. Participants were encouraged to integrate with the app daily, although it was not a requirement to do so.

**Figure 1.**
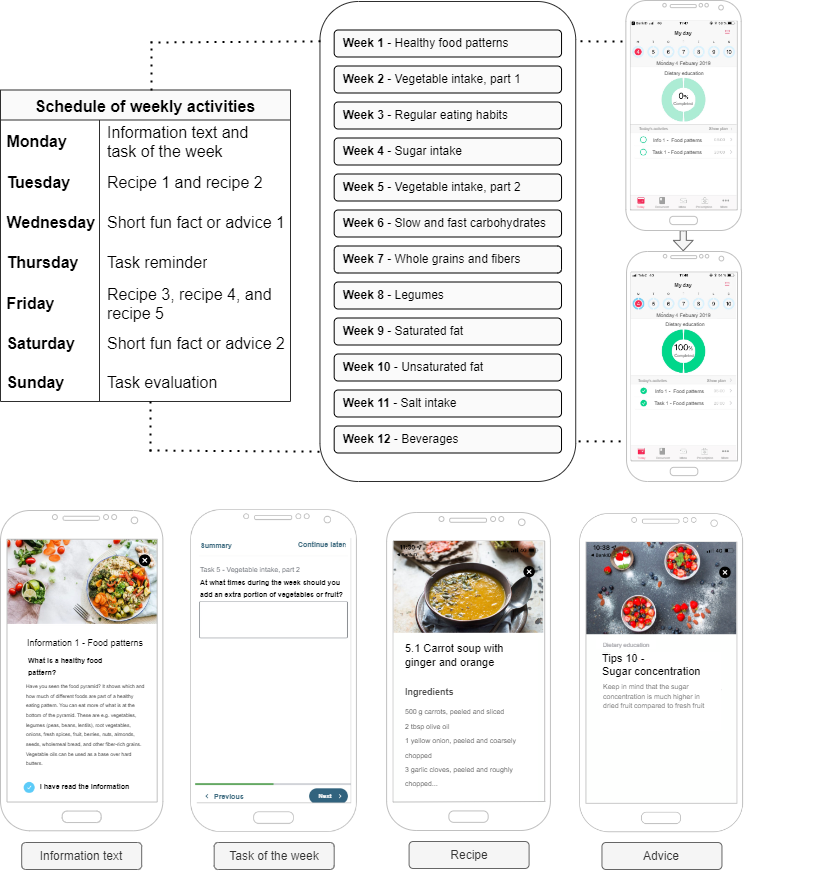
Overview of the weekly activity schedule, the topics of the 12 wk education in the randomized controlled HAPPY trial, and some examples of different features of the smartphone application: an educational information text, a weekly task, a recipe and advice.

The app has six different features, which included (1) educational information, (2) task introduction including a self-set goal of the week, (3) healthy recipes, (4) short fun facts or practical advice, (5) task reminder as a push notification, and (6) task evaluation “how did it go?” with the weekly task. A new topic was introduced each week, for example, “Healthy food patterns” or “Vegetable intake.” Figure 1 shows the 12 topics and gives examples of screens (here translated from Swedish to English) from the app. Each week followed the same activity schedule and included either 11 (weeks 3‐7 and weeks 10‐12) or 12 (weeks 1, 2, 8, and 9) activities depending on whether there were 4 or 5 recipes. The full 12-week course comprised 136 activities.

Users were able to follow their progress and track their percentage of completed activities in the app (see Figure 1). Before an activity is completed, 0% is visualized in the green circle, when all activities are completed for the day, the circle fills up and shows 100% completed activities. Uncompleted tasks were moved to the next day until marked as completed by the user. Information texts, advice, recipes etc, were saved in the app and could be accessed at any time during the study.

### Assessment of User Engagement

For the purposes of this study, data on completed activities was extracted from the app. We calculated an overall user engagement score in percent by summarizing all completed activities by a participant divided by the total number of activities. We also calculated the percentage of completed activities within each activity type. Based on the distribution of the overall engagement score, participants were categorized into groups of low (<50%), moderate (50‐99.9%), and high (100%) user engagement.

### Assessment of Dietary Intake

Participants dietary intake were assessed using a validated 95-items semiquantitative food frequency questionnaire (FFQ) [21,22] at baseline and after 3, 6 and 12 months of follow-up. Information on how often each item was consumed (eg once per day, week, or month) was reported by the participants. We obtained standard portions sized from the Swedish Food Composition Database from the National Food Agency [23] to calculate average daily intakes in grams of foods and beverages or grams or micrograms for nutrients.

We assessed overall dietary quality using a Nordic Nutrition Recommendations (NNR)-score based on the food-based dietary guidelines from the latest edition of the NNR 2023 [24]. We have described the NNR-score in detail previously [14]. The NNR-score components and the recommended intakes are presented in Multimedia Appendix 1 (see Table S1). In brief, our NNR-score was calculated based on 10 food based dietary guidelines presented in the NNR including vegetables, fruit and berries, whole grains (cereals), legumes, nuts and seeds, fish and seafood, red meat, vegetable oils, sweets including sugar-sweetened beverages (excluding salty snacks), and alcohol. Proportion scores ranged from 0‐3 points for each dietary guideline, with adherence complying to the recommendation yielding 3 points. This resulted in a total NNR score from 0 to 30 points. Furthermore, we examined dietary variables corresponding to the weekly topics of the dietary course (Figure 1).

### Background Characteristics

Background characteristics included age, sex (female and male), education level (≤12 years and >12 years), diabetes duration (<1 year, 1‐5 y, and >5 years), smoking status (never or former, and current), self-reported medication for hypertension, hyperlipidaemia, diabetes, and physical activity. Physical activity was assessed by 2 questions asking about time spent exercising and time spent performing daily activities, for example walking, cycling, or gardening, during a typical week [25]. A dichotomous variable (<150 min/week and ≥150 min/week) was created according to current physical activity recommendations [26]. We measured weight (kg), height (cm), and waist circumference (cm) and calculated BMI (kg/m^2^). BMI was further categorized as normal weight (<25 kg/m^2^), overweight (25.0‐29.9 kg/m^2^), and obesity (≥30.0 kg/m^2^) [27]. A dichotomous variable for waist circumference of low risk (<88 cm for women and<102 cm for men) and high risk (≥88 cm for women and ≥102 cm for men) for disease was also created [27]. We included baseline fasting HbA_1c_ (mmol/mol) as well as a dichotomous variable of HbA_1c_ (<52 mmol/mol and ≥52 mmol/mol), which is a general target value in the treatment of type 2 diabetes in Sweden [28].

### Statistical Analyses

Descriptive baseline characteristics were presented as mean (SD) continuous variables, as numbers (n), and percentages (%) for categorical variables. User engagement with the smartphone app was assessed by the proportion (%) of activities completed during the intervention period. We conducted analysis of variance to compare differences in continuous variables between user engagement groups, and *χ*^2^ tests for categorical data. Aspects of user engagement were also explored.

Paired *t* tests was used to compare mean changes in diet quality, dietary intake, and clinical markers within user engagement groups. We then fitted linear regression models to analyze differences in changes from baseline to the short-term and from baseline to the long-term follow-up between user engagement groups, with the low user engagement group used as the reference category. Long-term follow-up was not addressed for clinical markers, as these were not measured at the 12-month follow-up. Models were adjusted for baseline values of each dietary variable to account for differences in baseline intakes [29]. Potential confounders were identified using a Directed Acyclic Graph (DAG; see Figure S1 in Multimedia Appendix 1), and we also controlled for age, sex, intervention allocation, education level, BMI, diabetes duration, and HbA_1c_ level. Mean differences in changes for short-term and long-term intakes between groups, represented by β-coefficients with 95% CI, were calculated. Normality of the resulting residuals of each model was visually assessed using histograms with normal curve overlay and Q-Q (quantile-quantile) plots. All were found approximately normally distributed except for the model of sugar-sweetened beverages. This was because almost no one reported intake of sugar-sweetened beverages, which created no variation and thus a violated model assumption. Therefore, we did not run statistical models to analyse difference in change for this variable. Statistical analyses were performed using Stata (version 17.0; Stata Corporation), with the statistical significance level set at *P*<.05.

## Results

**Figure 2.**
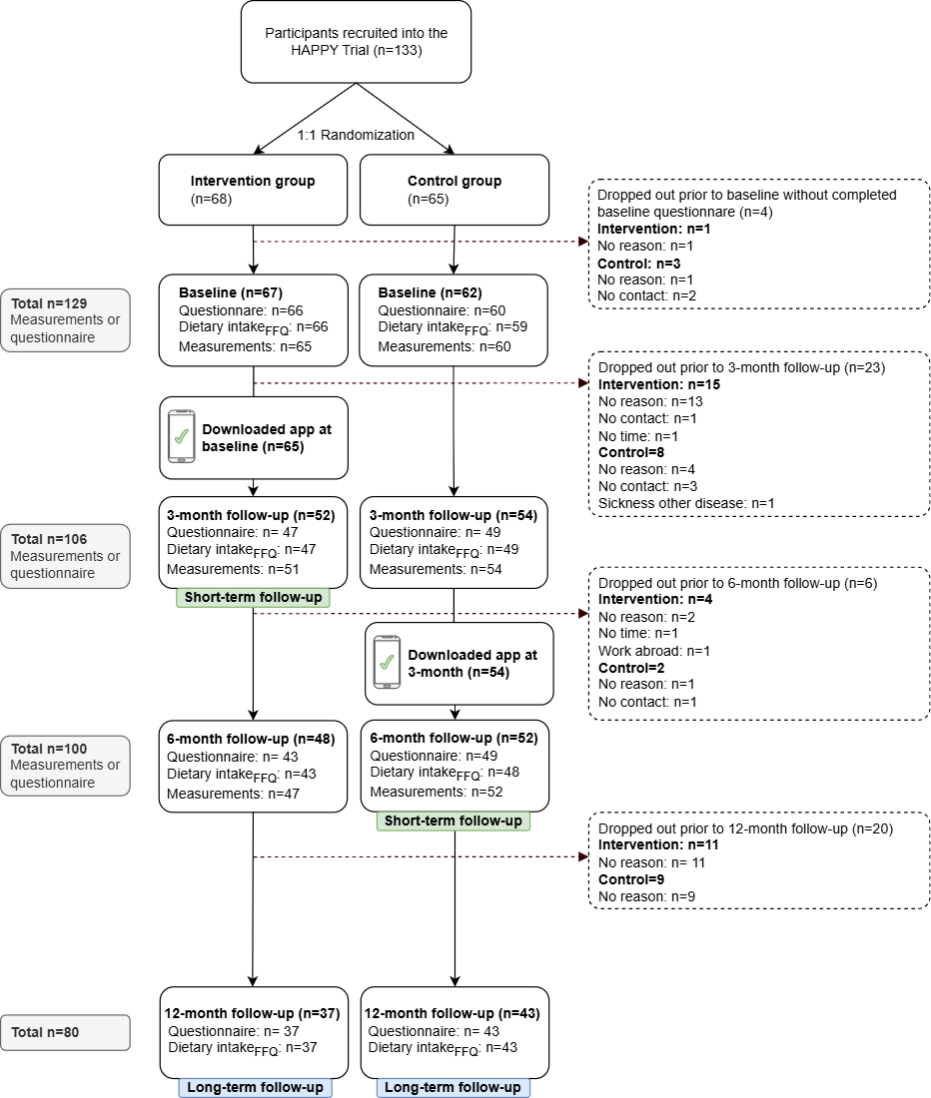
Flow diagram of persons with type 2 diabetes recruited from five primary care centers in Stockholm, Sweden, included in the randomized controlled HAPPY trial from recruitment and randomization to baseline, 3, 6, and 12 months follow-up. FFQ: Food Frequency Questionnaire; HAPPY: Healthy eating using APP technologY.

Figure 2 shows the CONSORT flow diagram of study participants through the trial. The baseline characteristics among all (n=119), and stratified by low (n=29), moderate (n=26), and high (n=64) app user engagement are shown in Table 1. The majority of study participants (60.5%, 72/119) were men, and the average age was 63.2 (SD 10.3) years. The mean BMI was 30.1 (SD 5.1) kg/m^2^, and most participants (61.2%, 71/116) had more than 12 years of education. Participants generally reported high levels of physical activity and 76.9% (90/112) met the recommended level of 150 min/week. Only 4/116 (3.5%) were current smokers. The mean HbA_1c_ level was 49.7 (SD 10.6) mmol/mol. Participants in the high user engagement group had a significantly higher NNR adherence score (mean 14.0, SD 3.4; *P*=.03) compared with low and moderate adherers (mean 12.2, SD 3.4 and mean 12.6, SD 2.8; respectively). Similarly, a statistically higher intake in fruit and vegetables (*P*=.04) was also seen in the high comparted with the other groups, while the moderate group had statistically higher intake of fish and seafood (*P*=.03) compared with the low and high adherers. There were no other statistically significant differences in baseline characteristics between user engagement groups.

**Table 1.** Baseline characteristics of study participants from the randomized controlled Healthy eating using APP technologY (HAPPY) trial that downloaded the app, all and by category of user engagement.

		User engagement^a^			
Variable	All (n=119)	Low (n=29)	Moderate (n=26)	High (n=64)	*P* value^b^
Characteristics, mean (SD)					
Age (years; n=119)	63.2 (10.3)	62.0 (9.5)	62.7 (11.4)	63.9 (10.2)	.37
BMI (kg/m^2^; n=119)	30.1 (5.1)	30.5 (5.9)	31.1 (6.1)	29.4 (4.1)	.31
HbA_1c_ (mmol/mol; n=110)	49.7 (10.6)	51.4 (12.5)	50.0 (10.1)	49.0 (10.1)	.66
Physical activity (min/week; n=117)	278.1 (150.9)	275.6 (158.5)	266.0 (152.1)	284.1 (149.4)	.87
Dietary variables (n=117), mean (SD)					
NNR^c^ score (0‐30 points)	13.3 (3.3)	12.2 (3.4)	12.6 (2.8)	14.0 (3.4)	.03
Fruit and vegetables (g/day)	356.3 (236.1)	261.2 (127.8)	356.7 (198.6)	396.2 (273.4)	.04
Legumes/pulses (g/day)	36.4 (36.4)	30.5 (28.6)	30.1 (22.5)	41.5 (43.0)	.25
Total fish and seafood (g/week)	330.9 (201.3)	252.5 (202.2)	393.0 (203.2)	338.7 (192.5)	.03
Red and processed meat (g/week)	592.4 (428.0)	502.1 (317.5)	659.1 (329.3)	603.4 (497.2)	.40
Sugar-sweetened beverages (g/day)	8.8 (50.0)	15.3 (55.2)	23.9 (89.2)	0.0 (0.0)	.09
Fiber (g/day)	27.6 (12.2)	25.4 (10.4)	27.3 (10.6)	28.6 (13.5)	.52
Whole grains (g/day)	62.5 (34.)	60.3 (40.7)	62.4 (33.4)	63.4 (32.8)	.93
Carbohydrates (g/day)	204.1 (75.0)	202.5 (75.0)	210.6 (79.1)	202.1 (74.4)	.88
Saturated fat (g/day)	30.9 (12.2)	29.3 (16.0)	35.2 (8.4)	29.8 (11.5)	.12
Unsaturated fat (g/day)	46.7 (18.9)	42.1 (20.6)	51.8 (13.6)	46.6 (19.8)	.17
Sodium (mg/day)	2623.1 (877.1)	2428.4 (911.9)	2851.2 (884.9)	2612.5 (851.4)	.21
Sucrose (g/day)	30.2 (15.2)	27.1 (13.9)	32.9 (17.2)	30.5 (14.8)	.39
Total energy (kcal/day)	2038.7 (658.2)	1936.0 (769.8)	2215.5 (589.2)	2010.1 (630.2)	.27
Sex, n (%)					.36
Female	47 (39.5)	10 (34.5)	8 (30.8)	29 (45.3)	
Male	72 (60.5)	19 (65.5)	18 (69.2)	35 (54.7)	
Education level, n (%)					.37
≤12 years	45 (38.8)	8 (29.6)	9 (34.6)	28 (44.4)	
>12 years	71 (61.2)	19 (70.4)	17 (65.4)	35 (55.6)	
BMI category (kg/m^2^), n (%)					.51
18.5‐24.9	19 (16)	2 (6.9)	5 (19.2)	12 (18.8)	
25‐29.9	46 (38.7)	14 (48.3)	8 (30.8)	24 (37.5)	
≥30	54 (45.4)	13 (44.8)	13 (50)	28 (43.8)	
Waist circumference (cm), n (%)					.16
Low	31 (26.1)	11 (37.9)	4 (15.4)	16 (25)	
High	88 (74)	18 (62.1)	22 (84.6)	48 (75)	
HbA_1c_ (mmol/mol), n (%)					.43
Low, <52	71 (64.6)	15 (68.2)	14 (53.9)	42 (67.4)	
High, ≥52	39 (35.5)	7 (31.8)	12 (46.2)	20 (32.3)	
Current smoker (yes), n (%)	4 (3.5)	1 (3.7)	2 (8)	1 (1.6)	.33
Physical activity (min/week), n (%)					.63
<150	22 (23.1)	8 (29.6)	6 (23.1)	13 (20.3)	
≥150	90 (76.9)	19 (70.4)	20 (76.9)	51 (79.7)	
Diabetes duration, n (%)					.90
<1 year	27 (24.1)	6 (23.1)	5 (20)	16 (26.2)	
1‐5 years	41 (36.6)	10 (38.5)	11 (44)	20 (32.8)	
>5 years	44 (39.3)	10 (38.5)	9 (36)	25 (41)	
Medical use, yes, n (%)	73 (62.4)	22 (81.5)	14 (53.9)	37 (57.8)	.06
Medication for (yes)^d^, n (%)					
Hypertension	80 (68.4)	21 (77.8)	19 (73.1)	40 (62.5)	.30
Diabetes, insulin	21 (18)	7 (25.9)	5 (19.2)	9 (14.1)	.40
Diabetes, Metformin	88 (75.2)	23 (85.2)	16 (61.5)	49 (76.6)	.13
Hyperlipidemia	73 (62.4)	22 (81.5)	14 (53.9)	37 (57.8)	.06

a User engagement groups, low <50%, moderate 50‐99.9%, high 100% of the total number of completed activities in the app.

b *P* value for difference between user engagement groups using analysis of variance for continuous variables and chi2 for categorical variables.

c NNR, Nordic Nutrition Recommendations.

d self-reported.

**Figure 3.**
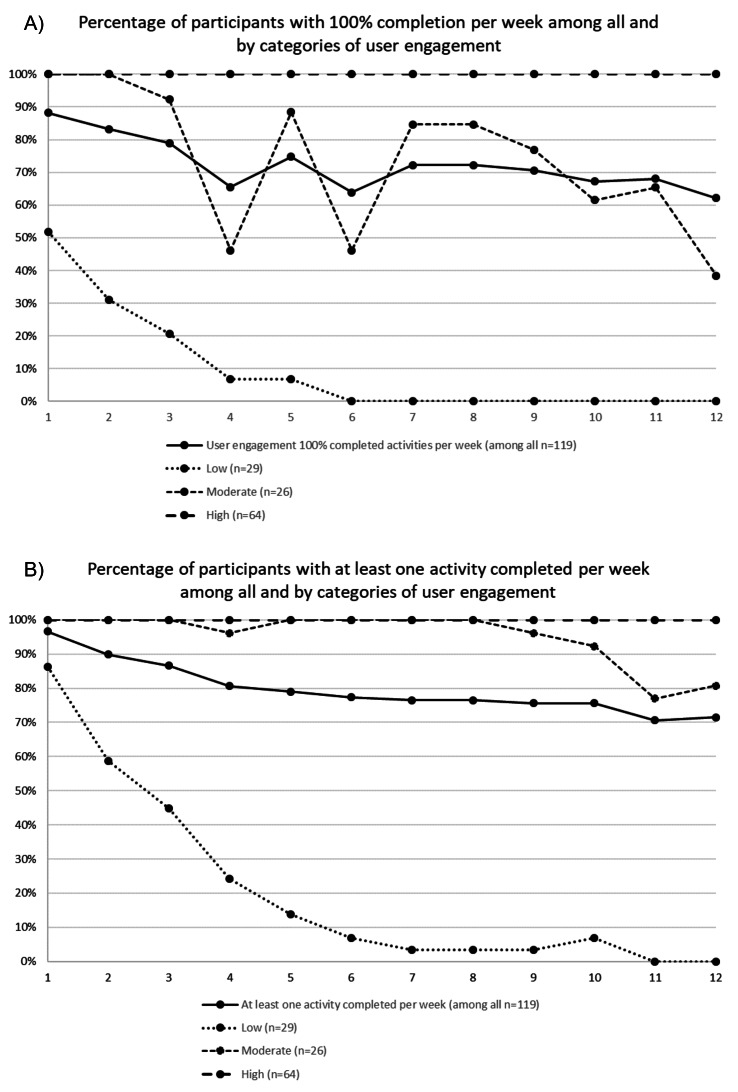
Proportion of persons with type 2 diabetes in the randomized controlled Healthy Eating using APP technologY (HAPPY) trial completing (1) all of the activities per week during the 12-week education and (2) at least one activity per week among all and by the categories of use engagement.

Overall, user engagement was high, with participants completing an average of 104.9 (77.1%) of the 136 activities in the app. Participants in the low, moderate and high user engagement groups completed on average 20.6 (15.1%), 121.9 (89.6%), and 136 (100%) of all activities, respectively. Among all participants, 88.2% (105/119) completed every activity during the first week, while 62.2% (74/119) completed every activity during the last week (see Figure 3A) and 71.4% (85/119) completed at least one activity per week throughout the 12 weeks (see Figure 3B).

Per definition, participants in the high user engagement group completed 100% of the activities each of the 12 wk (Figure 3A). Among moderate users, the proportion of participants completing 100% of the weekly activities was 100% (26/26) the first and 38.5% (10/26) the last week (Figure 3A). Nevertheless, during the last week, 80.8% (21/26) had completed at least one activity (Figure 3). Among participants with low user engagement, 51.2% (15/29) completed 100% of the weekly activities and 86.2% (25/29) completed at least one activity during the first week. Usage then rapidly decreased, and from week 6 and onwards, no one completed all activities.

**Figure 4.**
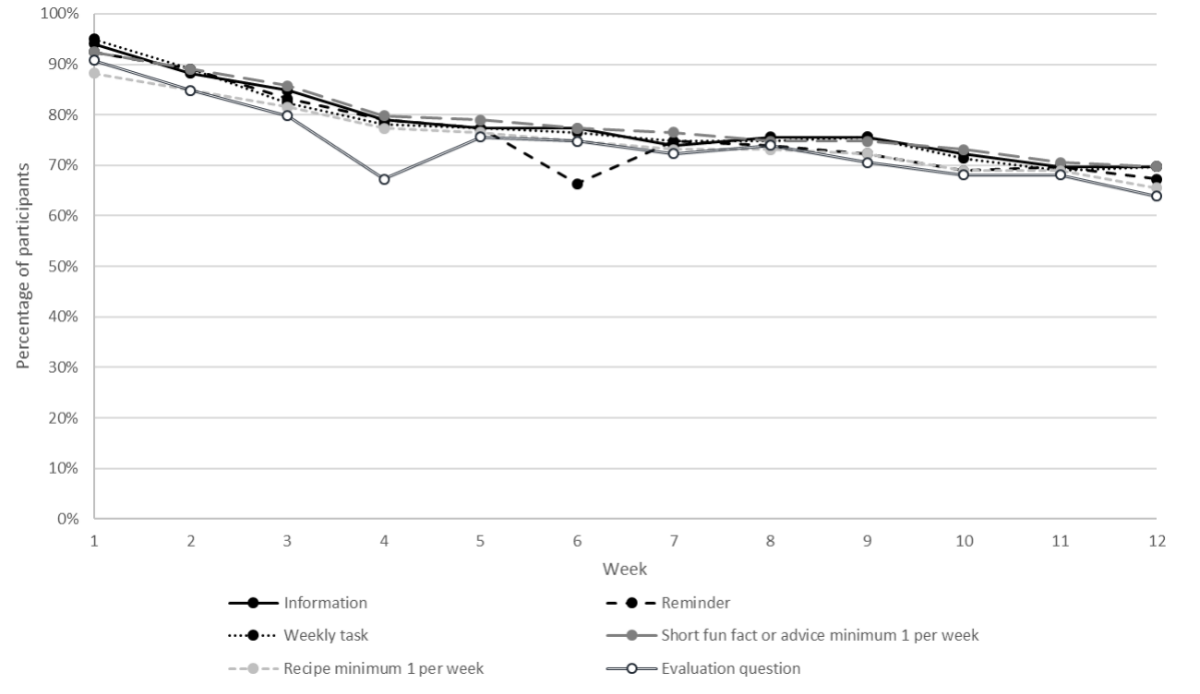
Proportion of persons with type 2 diabetes in the randomized controlled Healthy Eating using APP technologY (HAPPY) trial with completed activities, ie, active users, separated by the 6 different activity types among all participants throughout the 12 weeks.

Figure 4 shows the user engagement of the different activity types, that is, app features. The activity most often completed during the full 12 weeks was the short fun fact or advice (on average 78.6%, 93.5/119) of participants completed this activity every week), followed by educational information (78.2%, 93/119), the weekly task (77.8%, 92.6/119), the reminder of weekly task (76.2%, 90.7/119), recipes (75.4%, 89.4/119), and the evaluation question linked to the weekly task (74.2%, 88.3/119).

Table 2 presents the comparison of mean dietary intake levels within groups at baseline and the short-term follow-up, that is, directly after completed education, as well as results from linear regression models. The low user engagement group had a statistically significant decrease in whole grains (*P*=.03). Within the moderate user engagement group, a significant increase was seen in the NNR score (*P*=.03), and for fruit and vegetables (*P*=.04) and legumes (*P*=.04).

**Table 2.** Mean values and differences of short-term effects (directly after the 12-week dietary education) stratified by low, moderate and high app user engagement and linear regression models presented as difference in changes (β-coefficients and 95% CI) between the user engagement groups in the randomized controlled Healthy eating using APP technologY (HAPPY) trial. User engagement groups, low <50%, moderate 50%‐99%, high 100% of the total number of completed activities in the app.

								Crude^a^		Adjusted^b^	
Variable	Baseline		Short-term follow-up		Within change	Mean difference		Model estimates		Model estimates	
	Mean	(SD)	Mean	(SD)	*P* value^c^	Mean	(SD)	β	(95% CI)	β	(95% CI)
NNR^d^ (0‐30 points)											
Low user engagement	13.2	(2.3)	13.2	(2.6)	1.00	0.0	(2.7)	1.00	(reference)	1.00	(reference)
Moderate user engagement	12.2	(2.9)	13.7	(4.2)	.03	1.5	(3.1)	1.3	(-0.8 to 3.3)	1.4	(-0.7 to 3.8)
High user engagement	14.0	(3.3)	14.4	(3.6)	.22	0.5	(2.8)	0.6	(-1.2 to 2.5)	0.3	(-1.7 to 2.3)
Dietary variables (FFQ)^e^											
Fruit and vegetables (g/day)											
Low user engagement	297.8	(121.2)	279.5	(117.9)	.46	-18.2	(77.6)	1.00	(reference)	1.00	(reference)
Moderate user engagement	313.5	(155.7)	373.2	(189.0)	.04	59.7	(126.3)	84.2	(-11.7 to 180.2)	80.0	(-26.2 to 168.2)
High user engagement	391.2	(270.9)	402.5	(202.6)	.62	11.3	(180.5)	67.0	(-18.6 to 152.7)	31.1	(-60.1 to 122.4)
Legumes and pulses (g/day)											
Low user engagement	31.1	(33.2)	31.9	(35.0)	.79	0.8	(9.8)	1.00	(reference)	1.00	(reference)
Moderate user engagement	25.5	(19.4)	38.0	(35.3)	.04	12.5	(26.9)	8.9	(-19.9 to 37.7)	4.9	(-25.9 to 35.6)
High user engagement	42.4	(43.4)	46.8	(47.4)	.49	4.4	(50.4)	9.1	(-16.6 to 34.7)	9.3	(-19.3 to 38.0)
Total fish and seafood (g/week)											
Low user engagement	291.9	(205.8)	276.3	(147.7)	.76	-15.6	(163.6)	1.00	(reference)	1.00	(reference)
Moderate user engagement	379.9	(210.5)	327.3	(160.0)	.18	-52.6	(176.6)	-5.0	(-131.7 to 121.7)	-29.4	(-161.4 to 102.7)
High user engagement	338.9	(194.8)	348.3	(236.2)	.70	9.5	(191.4)	42.1	(-69.6 to 153.8)	6.1	(-116.9 to 129.2)
Red and processed meat (g/week)											
Low user engagement	602.8	(260.0)	632.3	(460.0)	.80	29.5	(377.5)	1.00	(reference)	1.00	(reference)
Moderate user engagement	661.3	(326.8)	662.3	(396.1)	.99	1.0	(469.4)	12.2	(-217.2 to 241.6)	4.8	(-225.7 to 235.4)
High user engagement	604.5	(502.9)	545.5	(291.5)	.29	-59.0	(438.1)	-87.3	(-290.4 to 115.8)	-90.9	(-308.0 to 126.2)
Sugar-sweetened beverages (g/day)											
Low user engagement	0.0	(0.0)	56.4	(133.7)	.19	56.4	(133.7)	-—^f^	-—	-—	-—
Moderate user engagement	28.2	(96.7)	28.2	(96.7)	1.00	0.0	(63.8)	-—	-—	-—	-—
High user engagement	0.0	(0.0)	3.3	(26.3)	.32	3.3	(26.3)	-—	-—	-—	-—
Fiber (g/day)											
Low user engagement	28.4	(9.7)	24.8	(7.5)	.13	-3.6	(7.2)	1.00	(reference)	1.00	(reference)
Moderate user engagement	25.7	(9.9)	28.8	(10.3)	.06	3.1	(7.2)	5.6	(-0.55 to 11.7)	5.4	(-0.96 to 11.7)
High user engagement	28.4	(13.5)	30.2	(12.0)	.19	1.8	(10.7)	5.4	(-0.04 to 10.8)	4.9	(-1.02 to 10.8)
Whole grains (g/day)											
Low user engagement	70.0	(37.1)	55.0	(20.8)	.03	-15.0	(18.9)	1.00	(reference)	1.00	(reference)
Moderate user engagement	62.7	(35.7)	63.4	(33.8)	.90	0.8	(28.7)	13.8	(-5.9 to 33.6)	15.6	(-5.5 to 36.7)
High user engagement	63.2	(33.2)	68.5	(39.6)	.16	5.4	(29.3)	18.6	(1.04 to 36.1)	20.4	(0.57 to 40.3)
Carbohydrates (g/day)											
Low user engagement	226.8	(61.8)	202.4	(84.8)	.23	-24.4	(62.7)	1.00	(reference)	1.00	(reference)
Moderate user engagement	207.7	(75.8)	210.3	(73.2)	.82	2.6	(52.2)	22.1	(-18.7 to 62.9)	18.9	(-24.6 to 62.4)
High user engagement	202.3	(74.9)	213.7	(78.1)	.14	11.4	(59.5)	29.5	(-6.7 to 65.7)	23.3	(-17.9 to 64.5)
Saturated fat (g/day)											
Low user engagement	37.3	(17.7)	36.2	(18.7)	.76	-1.1	(11.4)	1.00	(reference)	1.00	(reference)
Moderate user engagement	34.4	(7.8)	34.5	(10.3)	.99	0.0	(12.7)	-0.26	(-7.9 to 7.4)	0.12	(-7.9 to 8.1)
High user engagement	29.5	(11.4)	30.9	(11.0)	.34	1.4	(11.5)	-1.3	(-8.2 to 5.6)	-2.0	(-9.7 to 5.8)
Unsaturated fat (g/day)											
Low user engagement	53.6	(23.4)	51.5	(21.6)	.53	-2.1	(10.6)	1.00	(reference)	1.00	(reference)
Moderate user engagement	49.7	(10.9)	49.8	(12.6)	.98	0.1	(12.2)	0.66	(-9.8 to 11.1)	0.92	(-10.6 to 12.5)
High user engagement	46.2	(19.9)	47.8	(19.3)	.47	1.6	(17.6)	0.90	(-8.4 to 10.2)	2.0	(-9.0 to 13.0)
Sodium (mg/day)											
Low user engagement	2838.0	(773.5)	2694.8	(692.4)	.38	-143.2	(514.3)	1.00	(reference)	1.00	(reference)
Moderate user engagement	2778.0	(833.8)	2758.2	(713.7)	.90	-19.8	(738.5)	93.5	(-360.4 to 547.4)	35.8	(-447.4 to 518.9)
High user engagement	2603.5	(863.7)	2725.9	(768.4)	.22	122.4	(779.8)	148.9	(-254.7 to 552.5)	44.5	(-418.8 to 507.9)
Sucrose (g/day)											
Low user engagement	31.3	(12.5)	31.6	(19.7)	.94	0.3	(14.7)	1.00	(reference)	1.00	(reference)
Moderate user engagement	31.9	(13.9)	32.5	(13.2)	.82	0.6	(11.9)	0.48	(-7.8 to 8.8)	-1.2	(-9.9 to 7.5)
High user engagement	30.4	(14.8)	31.2	(14.3)	.61	0.8	(12.0)	0.13	(-7.2 to 7.5)	-1.8	(-9.9 to 6.3)
Total energy (kcal/day)											
Low user engagement	2258.5	(739.9)	2096.8	(789.3)	.29	-161.7	(481.3)	1.00	(reference)	1.00	(reference)
Moderate user engagement	2161.3	(533.7)	2147.7	(545.6)	.90	-13.6	(486.6)	111.6	(-240.8 to 464.1)	89.0	(-292.1 to 470.0)
High user engagement	2003.3	(636.0)	2095.6	(608.6)	0.19	92.3	(554.7)	158.2	(-156.4 to 472.8)	106.3	(-258.9 to 471.5)

a Results from the linear regression model adjusted for baseline values of the specific outcome variable to account for differences at baseline.

b Results from the linear regression model adjusted for baseline values, age, sex, intervention group, education level, BMI categories, diabetes duration, and HbA_1c_.

c Results from the paired *t* test within the user engagement groups.

d NNR: Nordic Nutrition Recommendations.

e FFQ: food frequency questionnaire.

f Not available.

Results from the linear regression models (see Table 2) showed a statistically significant increase in intake of whole grains (β=20.4, 95% CI 0.57-40.3) in the high compared with the low user engagement group. Table 3 shows the results for cardiometabolic risk markers are presented. Statistically significant within group differences from baseline to the short-term follow-up in BMI was seen in the low (*P*=.03) and the high user engagement groups (*P*=.001), and on waist circumference within the moderate (*P*=.006) and high (*P*<.001) user engagement groups. After fitting linear regression models, no statistically significant differences in change were seen between groups for any cardiometabolic risk markers.

**Table 3.** Mean values and differences of short-term effects (directly after the 12-week dietary education) stratified by low, moderate or high app user engagement and linear regression models presented as difference in changes (β-coefficients and 95% confidence intervals) between the user engagement groups in the randomized controlled Healthy eating using APP technologY (HAPPY) trial. User engagement groups, low <50%, moderate 50%‐99%, high 100% of the total number of completed activities in the app.

								Crude^a^		Adjusted^b^	
Variable	Baseline		Short-term effect		Within change	Mean difference		Model estimates		Model estimates	
	Mean	(SD)	Mean	(SD)	*P* value ^c^	Mean	(SD)	β	(95% CI)	β	(95% CI)
Cardiometabolic risk markers											
BMI (kg/m^2^)											
Low user engagement	29.3	(4.7)	28.6	(4.8)	.03	-0.7	(1.1)	1.00	(reference)	1.00	(reference)
Moderate user engagement	31.3	(6.2)	31.0	(6.0)	.13	-0.4	(1.1)	.38	(-0.20 to 0.97)	.35	(-0.28 to 0.99)
High user engagement	29.4	(4.1)	29.1	(4.1)	.001	-0.3	(0.)	.37	(-0.13 to 0.88)	.44	(-0.13 to 1.0)
Waist circumference											
Low user engagement	103.7	(11.4)	102.2	(11.6)	.15	-1.5	(3.9)	1.00	(reference)	1.00	(reference)
Moderate user engagement	110.5	(14.5)	107.6	(13.8)	.006	-3.0	(4.8)	-1.02	(-3.60 to 1.55)	-.80	(-3.5 to 1.8)
High user engagement	104.2	(12.5)	102.1	(12.5)	<.001	-2.1	(3.6)	-.53	(-2.7 to 1.70)	.22	(-2.2 to 2.6)
Body fat (%)											
Low user engagement	30.5	(9.8)	29.7	(10.0)	.16	-0.8	(1.8)	1.00	(reference)	1.00	(reference)
Moderate user engagement	33.6	(10.4)	32.5	(10.0)	.15	-1.0	(3.3)	-.19	(-1.95 to 1.57)	-0.07	(-1.9 to 1.8)
High user engagement	32.2	(8.5)	32.7	(8.8)	.12	0.5	(2.3)	1.26	(-0.29 to 2.82)	.96	(-0.76 to 2.7)
HbA_1c_ (mmol/mol)											
Low user engagement	53.0	(10.5)	51.3	(6.2)	.37	-1.7	(5.3)	1.00	(reference)	1.00	(reference)
Moderate user engagement	51.8	(9.9)	51.0	(10.5)	.61	-0.8	(7.0)	.62	(-4.00 to 5.24)	1.4	(-3.3 to 6.1)
High user engagement	47.8	(9.7)	47.2	(9.5)	.42	-0.7	(6.1)	-0.20	(-4.46 to 4.05)	-.66	(-4.6 to 3.6)
Triglycerides (mmol/L)											
Low user engagement	1.5	(0.8)	1.5	(0.7)	.83	-0.0	(0.5)	1.00	(reference)	1.00	(ref)
Moderate user engagement	1.7	(1.1)	1.9	(1.6)	.41	0.2	(1.1)	.26	(-0.45 to 0.97)	.39	(-0.37 to 1.1)
High user engagement	1.5	(0.8)	1.6	(1.1)	.34	0.1	(0.8)	.14	(-0.50 to 0.79)	.17	(-0.52 to 0.88)
Total cholesterol (mmol/L)											
Low user engagement	3.5	(0.7)	3.9	(0.7)	.22	0.4	(0.8)	1.00	(reference)	1.00	(reference)
Moderate user engagement	4.4	(1.2)	4.3	(1.3)	.45	-0.1	(0.7)	-.22	(-0.87 to 0.42)	-.19	(-0.87)
High user engagement	4.1	(1.0)	4.2	(1.0)	.39	0.1	(0.9)	-.09	(-0.67 to 0.49)	-.05	(-0‐66 to 0.56)
LDL^d^ cholesterol (mmol/L)											
Low user engagement	2.1	(0.4)	2.1	(0.6)	.80	0.0	(0.5)	1.00	(reference)	1.00	(reference)
Moderate user engagement	2.4	(0.7)	2.2	(0.7)	.15	-0.2	(0.5)	-.11	(-0.57 to 0.35)	-0‐19	(-0.67 to 0.29)
High user engagement	2.3	(0.9)	2.2	(0.8)	.73	-0.0	(0.7)	-.01	(-0.43 to 0.40)	-.003	(-0.44 to 0.43)
HDL^e^ cholesterol (mmol/L)											
Low user engagement	1.1	(0.3)	1.1	(0.3)	.12	0.0	(0.1)	1.00	(reference)	1.00	(reference)
Moderate user engagement	1.2	(0.3)	1.2	(0.3)	.18	0.0	(0.2)	.01	(-0.12 to 0.13)	-.003	(-0.13 to 0.14)
High user engagement	1.3	(0.4)	1.4	(0.4)	.16	0.0	(0.2)	.00	(-0.12 to 0.12)	.005	(-0.12 to 0.13)
Blood pressure (mmHg)											
*** ***Systolic											
Low user engagement	135.8	(18.0)	137.0	(13.0)	.77	1.2	(15.8)	1.00	(reference)	1.00	(reference)
Moderate user engagement	138.5	(16.3)	138.4	(14.3)	.95	-0.2	(13.6)	-.11	(-8.4 to 8.19)	1.8	(-7.3 to 10.8)
High user engagement	133.3	(13.7)	130.7	(15.7)	.16	-2.6	(14.3)	-4.96	(-12.2 to 2.30)	-3.6	(-11.9 to 4.6)
Diastolic											
Low user engagement	83.3	(14.1)	85.9	(9.6)	.42	2.6	(12.0)	1.00	(reference)	1.00	(reference)
Moderate user engagement	88.3	(11.3)	87.6	(12.3)	.69	-0.7	(7.9)	-1.28	(-6.99 to 4.42)	-1.0	(-7.5 to 5.4)
High user engagement	82.6	(11.2)	82.2	(10.9)	.76	-0.4	(9.9)	-3.27	(-8.22 to 1.68)	-2.0	(-7.9 to 3.9)

a Results from the linear regression model adjusted for baseline values of the specific outcome variable to account for differences at baseline.

b Results from the linear regression model adjusted for baseline values, age, sex, intervention group, education level, BMI categories, diabetes duration, and HbA_1c_.

c Results from the paired *t* test within the user engagement groups.

d LDL: low-density lipoprotein.

e HDL: high-density lipoprotein.

Table 4 shows the results from the long-term follow-up. When comparing within group differences, the moderate user engagement group had a statistically significant higher intake of fiber (*P*=.004) and whole grains (*P*=.01) at the long-term follow-up compared to baseline. No other significant differences were seen within the user engagement groups.

In linear regression models (Table 4), a statistically significant difference in change in fiber (β=7.3, 95% CI 1.2-13.4) and whole grain intake (β=26.6, 95% CI 2.4-50.8) was seen in the moderate engagement group compared with the low user engagement group. Among those with moderate user engagement compared to those with low, statistically significant differences in change were also seen for saturated fat (β=11.7, 95% CI 2.9-20.5), sodium (β=632.0, 95% CI 95.0-1169.0), and total energy (β=568.1, 95% CI 133.0-1003.1).

**Table 4.** Mean values and differences of long-term effects (9 months after the 12-week dietary education in the intervention group and 6 months after the education in the control group) stratified by low, moderate and high app user engagement and linear regression models presented as difference in changes (β-coefficients and 95% confidence intervals) between the user engagement groups in the randomized controlled Healthy eating using APP technologY (HAPPY) trial. User engagement groups, low <50%, moderate 50%‐99.9%, high 100% of the total number of completed activities in the app.

								Crude^a^		Adjusted^b^	
Variable	Baseline		Long-term effect		Within change	Mean difference		Model estimates		Model estimates	
	Mean	(SD)	Mean	(SD)	*P* value^c^	Mean	(SD)	β	(95% CI)	β	(95% CI)
NNR^d^ (0‐30 points)											
Low user engagement	12.7	(2.9)	13.5	(3.1)	.52	0.7	(3.6)	1.00	(reference)	1.00	(reference)
Moderate user engagement	12.1	(3.2)	13.4	(3.9)	.07	1.4	(2.8)	0.4	(-1.9 to 2.7)	0.8	(-1.7 to 3.3)
High user engagement	13.9	(3.3)	14.0	(3.6)	.72	0.2	(3.1)	-0.2	(-2.1 to 1.8)	-0.9	(-3.2 to 1.4)
Dietary variables (FFQ)^e^											
Fruit and vegetables (g/day)											
Low user engagement	279.1	(129.0)	267.3	(95.3)	.71	-11.8	(103.8)	1.00	(reference)	1.00	(reference)
Moderate user engagement	316.6	(144.7)	375.0	(375.0)	.08	58.5	(125.5)	85.5	(-23.1 to 194.2)	49.0	(-62.5 to 160.6)
High user engagement	390.3	(264.6)	397.8	(208.0)	.77	7.5	(186.5)	64.5	(-28.6 to 157.6)	29.3	(-70.3 to 128.8)
Legumes/pulses (g/day)											
Low user engagement	32.9	(32.6)	27.8	(37.9)	.52	-5.1	(25.6)	1.00	(reference)	1.00	(reference)
Moderate user engagement	24.4	(19.1)	38.2	(45.2)	.18	13.7	(39.4)	14.2	(-8.7 to 37.2)	11.2	(-14.9 to 37.3)
High user engagement	39.2	(37.5)	32.5	(27.3)	.17	-6.7	(34.5)	1.88	(-17.5 to 21.3)	0.57	(-22.3 to 23.5)
Total fish and seafood (g/week)											
Low user engagement	261.3	(212.7)	240.2	(97.1)	.63	-21.1	(139.0)	1.00	(reference)	1.00	(reference)
Moderate user engagement	365.0	(225.8)	295.5	(182.8)	.18	-69.5	(195.6)	9.02	(-135.2 to 153.3)	29.8	(-126.2 to 185.8)
High user engagement	348.3	(195.5)	343.4	(223.0)	.88	-4.9	(230.4)	64.4	(-57.6 to 186.4)	67.0	(-71.5 to 205.4)
Red and processed meat (g/week)											
Low user engagement	559.3	(302.3)	576.4	(363.5)	.84	17.0	(266.5)	1.00	(reference)	1.00	(reference)
Moderate user engagement	681.0	(355.7)	682.9	(355.6)	.98	1.9	(259.8)	62.6	(-169.8 to 295.0)	61.2	(-188.8 to 311.2)
High user engagement	595.9	(513.1)	548.8	(329.4)	.47	-47.1	(474.7)	-40.8	(-236.9 to 155.3)	-61.2	(-283.7 to 161.4)
Sugar-sweetened beverages (g/day)											
Low user engagement	0.0	(0.0)	18.8	(62.4)	.34	18.8	(62.4)	—^g^	—	—	—
Moderate user engagement	25.9	(103.4)	25.9	(103.4)	—^f^	0.0	(0.0)	—	—	—	—
High user engagement	0.0	(0.0)	7.8	(56.8)	.32	7.8	(56.8)	—	—	—	—
Fiber (g/day)											
Low user engagement	28.4	(9.9)	24.7	(9.8)	.15	-3.7	(8.0)	1.00	(reference)	1.00	(reference)
Moderate user engagement	25.4	(9.7)	30.4	(13.9)	.004	5.0	(5.8)	7.9	(2.2 to 13.6)	7.3	(1.2 to 13.4)
High user engagement	27.9	(13.5)	29.0	(11.3)	.35	1.1	(8.5)	4.7	(-0.12 to 9.6)	3.9	(-1.5 to 9.3)
Whole grains (g/day)											
Low user engagement	74.2	(35.3)	60.0	(37.2)	.12	-14.1	(27.3)	1.00	(reference)	1.00	(reference)
Moderate user engagement	55.3	(28.5)	69.5	(36.7)	.01	14.2	(19.4)	24.4	(3.1 to 45.8)	26.6	(2.4 to 50.8)
High user engagement	60.0	(31.9)	66.0	(36.6)	.15	6.0	(29.5)	17.2	(-0.79 to 35.1)	18.2	(-3.2 to 39.6)
Carbohydrates (g/day)											
Low user engagement	236.9	(78.4)	192.4	(77.9)	.07	-44.5	(71.3)	1.00	(reference)	1.00	(reference)
Moderate user engagement	212.2	(72.2)	230.8	(89.9)	.17	18.7	(52.2)	56.4	(14.1 to 98.7)	60.9	(13.8 to 108.1)
High user engagement	201.4	(79.3)	209.7	(74.1)	.29	8.3	(56.2)	43.1	(7.1 to 79.2)	46.2	(3.6 to 88.7)
Saturated fat (g/day)											
Low user engagement	39.2	(17.2)	31.5	(8.3)	.06	-7.7	(11.8)	1.00	(reference)	1.00	(reference)
Moderate user engagement	35.5	(8.1)	39.2	(14.5)	.36	3.7	(15.6)	9.8	(2.2 to 17.3)	11.7	(2.9 to 20.5)
High user engagement	29.4	(12.0)	30.4	(11.5)	.40	1.0	(9.0)	4.4	(-2.2 to 11.1)	5.9	(-2.3 to 14.1)
Unsaturated fat (g/day)											
Low user engagement	54.0	(24.4)	53.0	(16.0)	.82	-1.1	(15.3)	1.00	(reference)	1.00	(reference)
Moderate user engagement	49.6	(12.4)	54.9	(22.4)	.35	5.3	(21.7)	4.8	(-7.5 to 17.0)	6.9	(-7.2 to 21.0)
High user engagement	45.4	(19.5)	48.0	(19.5)	.23	2.6	(15.8)	0.54	(-9.9 to 11.0)	2.3	(-10.5 to 15.3)
Sodium (mg/day)											
Low user engagement	2837.7	(895.1)	2549.5	(795.8)	.23	-288.2	(742.9)	1.00	(reference)	1.00	(reference)
Moderate user engagement	2776.9	(906.3)	3064.4	(1136.0)	.13	287.5	(719.9)	550.9	(58.6 to 1043.1)	632.0	(95.0 to 1169.0)
High user engagement	2595.6	(869.2)	2618.8	(698.7)	.82	23.2	(719.6)	212.4	(-205.8 to 630.6)	210.7	(-277.9 to 699.3)
Sucrose (g/day)											
Low user engagement	31.6	(14.2)	28.0	(13.9)	.18	-3.6	(8.3)	1.00	(reference)	1.00	(reference)
Moderate user engagement	31.7	(13.1)	34.2	(17.0)	.45	2.5	(12.8)	6.1	(-2.2 to 14.4)	4.9	(-4.4 to 14.3)
High user engagement	31.0	(15.5)	31.6	(13.8)	.73	0.6	(11.9)	4.0	(-3.1 to 11.0)	2.6	(-5.9 to 11.1)
Total energy (kcal/day)											
Low user engagement	2370.4	(867.1)	1971.4	(588.6)	.06	-399.0	(628.1)	1.00	(reference)	1.00	(reference)
Moderate user engagement	2181.2	(544.8)	2350.7	(785.2)	.28	169.5	(609.1)	496.2	(114.3 to 878.0)	568.1	(133.0 to 1003.1)
High user engagement	1989.1	(655.4)	2062.9	(596.0)	.30	73.8	(511.2)	327.1	(-1.03 to 655.2)	391.0	(-10.3 to 792.4)

a Results from the linear regression model adjusted for baseline values of the specific outcome variable to account for differences at baseline.

b Results from the linear regression model adjusted for baseline values, age, sex, intervention group, education level, BMI categories, diabetes duration and HbA_1c_.

c Results from the paired *t* test within the user engagement groups.

d NNR: Nordic Nutrition Recommendations.

e FFQ: food frequency questionnaire.

f Too little variation in data.

g Not available.

## Discussion

### Principal Findings

Our results showed that user engagement of a 12-week app-based dietary education targeting people with type 2 diabetes was high. Those with the highest user engagement followed the NNR guidelines to a greater extent and had higher intake of fruit and vegetables already at baseline. High user engagement was associated with beneficial changes in whole grain intake in the short term, that is, directly following the intervention. We found no associations between user engagement and cardiometabolic risk markers. Long-term participants with moderate user engagement continued to report an increase in whole grain intake, along with additional beneficial changes in fiber intake and an increased intake of carbohydrates 6 to 9 months postintervention in comparison to participants with low user engagement. Interestingly, the moderate group had a higher intake of total energy, saturated fat, and sodium at the long-term follow-up, resulting in a significant difference in change compared with the low user engagement group. Thus, the low user engagement group had a comparatively better diet in terms of saturated fat and sodium intake.

Despite the importance of addressing user engagement, studies commonly do not mention this when presenting their results [9,30-32]. The comparison of studies is further complicated by the lack of a standardized definition. Sittig et al [33] reported high user engagement in a 9-week app-based lifestyle intervention, with participants completing, 75.1% of 177 activities, similar to our 77.1%. Their app, like ours, included written educational content and recipes, but also short educational videos. The length of their intervention was also relatively similar, 9 vs 12 weeks, respectively. In another study by Mummah et al [34], 75% (51/68) of participants engaged at least once with their app targeting vegetable consumption during 8 weeks. Low user engagement was reported by Alonso-Domínguez et al [35], with average app use being only 35 days (corresponding to 38.9% of 90 days) during a 3-month multicomponent diet intervention. Data on user-engagement for longer durations are limited, but Lim et al [36] showed that 62% (61/99) of participants used at least one app feature on at least 75% of days during 6 months. Among motivated participants, maintaining high user engagement for longer time periods may be feasible.

Lavikainen et al [12] identified 4 user engagement patterns during a 12-month app-based lifestyle intervention. They found that 46.9% (904/1926) discontinued use, 38% (731/1926) used the app weekly, 10.8% (208/1926) twice a week, and 4.3% (83/1926) daily. Similar to our findings, high user engagement was associated with improved diet quality, and scoring high on a Healthy Diet Index at baseline increased the odds of belonging to a high user engagement group. This suggests that those motivated to use the app already have a healthier diet. Furthermore, Mummah et al [34] showed that baseline vegetable consumption was a moderator of effect and the effect of their intervention increased with baseline consumption. Hendrie et al [13] showed that baseline vegetable consumption and app use were the best predictors of improved variety and intake of vegetables. Interestingly in this case, those with lower baseline intakes increased their variety most and participants with the highest app use had almost doubled their intake compared to participants with the lowest use. Hence, app effectiveness may relate to both app-use and participant characteristics.

We observed a decrease in completed activities over time for the moderate and low user engagement groups, similar to previous mHealth interventions [37-39]. Nevertheless, the majority, over 70%, of our participants completed at least one activity every week. Somewhat fewer participants engaged with the app during weeks targeting sugar intake and slow and fast carbohydrates. One of the most common topics in diabetes management is carbohydrate calculation [40] and potentially our participants felt confident in this, resulting in less engagement. This is in contrast to previous research showing that user engagement can be improved by focusing on health problems or specific needs [10].

Future studies need to investigate reasons for why people stop engaging with apps. Declining app use has been identified as an early indicator of disengagement in a digital lifestyle intervention, highlighting the importance of closely monitoring and support users during the initial phase [12]. Components to increase user engagement in apps, including commercial apps, include gamification (eg, goal setting and quizzes), a more personal approach to app content, such as individualized and tailored reminders, automated data collection, and human-like features [4,41]. A user-friendly design, culturally and personally tailored content, and caregiver support have also been found to play a role [42]. In our app-design, we used gamification elements, such as allowing the user to set a personal goal for the week and progress-track the percentage of activities that had been completed each day, which has been shown to serve positive reinforcement for behavior changes [42]. This, hopefully, encouraged users to be more active in their own progress. However, our app did not include personalized reminders, for example, through timing where the user can set customized reminders based on their needs, which might have increased engagement.

Previous app experiences and social support within apps, such as networking and experience sharing, have been shown to strengthen user engagement, but while social support may influence behavior change for some, it can also introduce negative social competition [42]. We did not include any social elements in our app. In a qualitative study, people with type 2 diabetes preferred app features that supported them to make informed and independent decisions about their own care, rather than following explicit directives [41]. Factors that could make our app more personal and, thus, more engaging could include allowing participants to choose specific activities in the app, such as only receiving educational information, or receiving recipes suitable for their preferences, such as a vegetarian diet.

### Strengths and Limitations

A strength of this study is that we automatically collected detailed data on app usage. However, we do not know how much time users spent with the app. It is a limitation that we did not explore why user engagement dropped over time. The inclusion of both women and men is another strength, especially since men are usually more challenging to recruit in interventions [43]. The sex distribution and other characteristics, such as age, BMI, and HbA_1c_ levels, in our study are similar to the average adult population with type 2 diabetes in Swedish primary care [44]. Among our participants, 84.2% were overweight or obese, compared to 82.7% in primary care. However, our participants were slightly younger (63.2 years vs 68.7 years) and had a lower HbA_1c_ (47.7 mmol/mol vs 52.0 mmol/mol). The fact that participants were recruited in primary care and from areas with relatively high socioeconomic status, and that the app was only available in Swedish, limits the generalizability of our results to other socioeconomic groups.

Additional strengths include use of a validated FFQ [21,22] and addressing both short-term and long-term dietary intake. This allowed us to examine if changes were maintained over time. Furthermore, anthropometrics and cardiometabolic risk markers were objectively measured, but the latter only at short-term follow-up. Different long-term follow-up intervals between participants in the two groups is also a limitation. In addition, power was calculated based on the primary outcome of the trial [15]. The small sample size in this study may have been underpowered to detect statistical significance in secondary analyses. Finally, social desirability bias and recall bias may have influenced reporting in the FFQ. For example, foods considered healthier may be over-reported and unhealthy foods more under-reported [45]. However, we believe this is likely to be random and not related to a specific user engagement group.

### Conclusion

In our study, engagement with a smartphone-based dietary education was high, suggesting that an app targeting dietary habits is feasible to use. Those with high user engagement maintained healthier dietary behaviors over time, compared to those with low user engagement. Future studies should address the challenge of designing targeted mHealth solutions to engage individuals in maintaining app-usage during the full extent of interventions.

## Supplementary material

10.2196/71408 Multimedia Appendix 1 Description of the Nordic Nutrition Recommendations 2023 (NNR) score and a directed acyclic graph (DAG) displaying potential confounders in the association between user engagement and dietary changes.

10.2196/71408 Checklist 1 CONSORT-EHEALTH checklist V 1.6.1.

## Abbreviations

CONSORT-EHEALTH: Consolidated Standards of Reporting Trials of Electronic and Mobile HEalth Applications and onLine TeleHealth
FFQ: Food Frequency Questionnaire
HAPPY: Healthy Eating using APP technologY
HDL: high-density lipoprotein cholesterol
LDL: low-density lipoprotein cholesterol
mHealth: mobile health
NNR: Nordic Nutrition Recommendations

## References

[1] Petroni ML, Brodosi L, Marchignoli F (2021). Nutrition in patients with type 2 diabetes: present knowledge and remaining challenges. Nutrients.

[2] Evert AB, Dennison M, Gardner CD (2019). Nutrition therapy for adults with diabetes or prediabetes: a consensus report. Diabetes Care.

[3] (2018). MHealth - use of appropriate digital technologies for public health. https://apps.who.int/gb/ebwha/pdf_files/WHA71/A71_20-en.pdf.

[4] Jakob R, Harperink S, Rudolf AM (2022). Factors influencing adherence to mhealth apps for prevention or management of noncommunicable diseases: systematic review. J Med Internet Res.

[5] Sieverink F, Kelders SM, van Gemert-Pijnen JE (2017). Clarifying the concept of adherence to eHealth technology: systematic review on when usage becomes adherence. J Med Internet Res.

[6] Donkin L, Christensen H, Naismith SL, Neal B, Hickie IB, Glozier N (2011). A systematic review of the impact of adherence on the effectiveness of e-therapies. J Med Internet Res.

[7] Perski O, Blandford A, West R, Michie S (2017). Conceptualising engagement with digital behaviour change interventions: a systematic review using principles from critical interpretive synthesis. Transl Behav Med.

[8] Yardley L, Spring BJ, Riper H (2016). Understanding and promoting effective engagement with digital behavior change interventions. Am J Prev Med.

[9] Young C, Campolonghi S, Ponsonby S (2019). Supporting engagement, adherence, and behavior change in online dietary interventions. J Nutr Educ Behav.

[10] Schubart JR, Stuckey HL, Ganeshamoorthy A, Sciamanna CN (2011). Chronic health conditions and internet behavioral interventions: A review of factors to enhance user engagement. Comput Inform Nurs.

[11] Kelders SM, Kok RN, Ossebaard HC, Van Gemert-Pijnen J (2012). Persuasive system design does matter: a systematic review of adherence to web-based interventions. J Med Internet Res.

[12] Lavikainen P, Mattila E, Absetz P (2022). Digitally supported lifestyle intervention to prevent type 2 diabetes through healthy habits: secondary analysis of long-term user engagement trajectories in a randomized controlled trial. J Med Internet Res.

[13] Hendrie GA, Hussain MS, Brindal E, James-Martin G, Williams G, Crook A (2020). Impact of a mobile phone app to increase vegetable consumption and variety in adults: large-scale community cohort study. JMIR Mhealth Uhealth.

[14] Sjöblom L, Hantikainen E, Dahlgren A, Trolle Lagerros Y, Bonn SE (2025). The effect of an app-based dietary education on dietary intake and cardiometabolic risk markers in people with type 2 diabetes: results from a randomized controlled trial. Nutr J.

[15] Trolle Lagerros Y, Dahlgren A, Sjöblom L, Bonn SE (2020). Digital support for healthier eating habits among patients with type 2 diabetes: protocol for a randomized clinical trial within primary care (HAPPY trial). JMIR Res Protoc.

[16] (2015). Find your way - the swedish dietary advice [hitta ditt sätt - de svenska kostråden]. The Swedish Food Agency [Livsmedelsverket].

[17] Champion VL, Skinner CS (2008). Health Behav Health Educ Theory Res Pract.

[18] Shaffer JA, Gellman MD, Turner JR (2013). Encycl Behav Med.

[19] Bandura A (1998). Health promotion from the perspective of social cognitive theory. Psychology & Health.

[20] National Cancer Institute US (2012). Department of Health and Human Services Theory at A Glance A Guide For Health Promotion Practice.

[21] Eke H, Sjöblom L, Lagerros YT, Bonn SE (2024). A validation study comparing energy and nutrient intake between A web-based food frequency questionnaire and A 4-d dietary record. Nutrition.

[22] Messerer M, Johansson SE, Wolk A (2004). The validity of questionnaire-based micronutrient intake estimates is increased by including dietary supplement use in Swedish men. J Nutr.

[23] (2023). Livsmedelsdatabasen [the swedish food composition database]. Livsmedelsverket [The Swedish Food Agency].

[24] Blomhoff R, Andersen R, Arnesen EK (2023). Nordic Nutrition Recommendations 2023.

[25] Olsson SJG, Ekblom Ö, Andersson E, Börjesson M, Kallings LV (2016). Categorical answer modes provide superior validity to open answers when asking for level of physical activity: A cross-sectional study. Scand J Public Health.

[26] (2022). Physical activity. World Health Organization.

[27] (2011). Waist circumference and waist-hip ratio: report of a WHO expert consultation. World Health Organization.

[28] (2022). Blood sample: HBA1c [blodprov: HBA1c]. 1177.

[29] Twisk J, Bosman L, Rijnhart J, Hoekstra T, Welten M, Heymans M (2018). Different ways to estimate treatment effects in randomised controlled trials. Contemp Clin Trials Commun.

[30] Lugones-Sánchez C, Recio-Rodríguez JI, Menéndez-Suárez M (2022). Effect of a multicomponent mHealth intervention on the composition of diet in a population with overweight and obesity-randomized clinical trial EVIDENT 3. Nutrients.

[31] Martin CK, Miller AC, Thomas DM, Champagne CM, Han H, Church T (2015). Efficacy of SmartLoss SM, a smartphone-based weight loss intervention: results from a randomized controlled trial. Obesity (Silver Spring).

[32] Sun C, Sun L, Xi S (2019). Mobile phone-based telemedicine practice in older Chinese patients with type 2 diabetes mellitus: randomized controlled trial. JMIR Mhealth Uhealth.

[33] Sittig S, Wang J, Iyengar S, Myneni S, Franklin A (2020). Incorporating behavioral trigger messages into a mobile health app for chronic disease management: randomized clinical feasibility trial in diabetes. JMIR Mhealth Uhealth.

[34] Mummah S, Robinson TN, Mathur M, Farzinkhou S, Sutton S, Gardner CD (2017). Effect of a mobile app intervention on vegetable consumption in overweight adults: a randomized controlled trial. Int J Behav Nutr Phys Act.

[35] Alonso-Domínguez R, García-Ortiz L, Patino-Alonso MC, Sánchez-Aguadero N, Gómez-Marcos MA, Recio-Rodríguez JI (2019). Effectiveness of a multifactorial intervention in increasing adherence to the mediterranean diet among patients with diabetes mellitus type 2: a controlled and randomized study (EMID study). Nutrients.

[36] Lim SL, Ong KW, Johal J (2021). Effect of a smartphone app on weight change and metabolic outcomes in Asian adults with type 2 diabetes: a randomized clinical trial. JAMA Netw Open.

[37] Gong E, Baptista S, Russell A (2020). My Diabetes Coach, a mobile app-based interactive conversational agent to support type 2 diabetes self-management: randomized effectiveness-implementation trial. J Med Internet Res.

[38] Lee EY, Cha SA, Yun JS (2022). Efficacy of personalized diabetes self-care using an electronic medical record-integrated mobile app in patients with type 2 diabetes: 6-month randomized controlled trial. J Med Internet Res.

[39] Nelson LA, Spieker A, Greevy R, LeStourgeon LM, Wallston KA, Mayberry LS (2020). User engagement among diverse adults in a 12-month text message-delivered diabetes support intervention: results from a randomized controlled trial. JMIR Mhealth Uhealth.

[40] Hood M, Wilson R, Corsica J, Bradley L, Chirinos D, Vivo A (2016). What do we know about mobile applications for diabetes self-management? A review of reviews. J Behav Med.

[41] Baptista S, Wadley G, Bird D, Oldenburg B, Speight J, My Diabetes Coach Research Group (2020). User experiences with a type 2 diabetes coaching app: qualitative study. JMIR Diabetes.

[42] Szinay D, Jones A, Chadborn T, Brown J, Naughton F (2020). Influences on the uptake of and engagement with health and well-being smartphone apps: systematic review. J Med Internet Res.

[43] Pagoto SL, Schneider KL, Oleski JL, Luciani JM, Bodenlos JS, Whited MC (2012). Male inclusion in randomized controlled trials of lifestyle weight loss interventions. Obesity (Silver Spring).

[44] Eeg-Olofsson K, Åkesson K, Nåtman J (2022). Nationella diabetesregistret, årsrapport 2022 [Article in Swedish]. https://www.ndr.nu/pdfs/Arsrapport_NDR_2022.pdf.

[45] Warnecke RB, Johnson TP, Chávez N (1997). Improving question wording in surveys of culturally diverse populations. Ann Epidemiol.

