# Supplementary material for: Engagement With a Smartphone-Delivered Dietary Education Intervention and Its Relation to Dietary Intake and Cardiometabolic Risk Markers in People With Type 2 Diabetes: Secondary Analysis of a Randomized Controlled Trial
Source: JMIR Form Res. 2025 May 30;9:e71408. doi: 10.2196/71408 (PMC12168189; doi:10.2196/71408)
Supplement: Multimedia Appendix 1 [file formative-v9-e71408-s001.docx]

## Multimedia Appendix 1

| **Supplemental Table S1.** Nordic Nutrition Recommendations 2023 (NNR) score based on the food-based dietary guidelines^a^ and cut-offs points for adherence. | | |
| --- | --- | --- |
| **NNR-score components** | **Recommended intake** | **Total NNR-score, 0-30 points (10 components, 0-3 points each)** |
| 1) Vegetables, fruits and berries, g/day | ≥500-800 | 0 points: <100  1 point: 100-299  2 points: 300-499  **3 points: ≥500** |
| 2) Cereals, whole grains, g/day | ≥90 | 0 points: <30  1 point: 30-59  2 points: 60-89  **3 points: ≥90** |
| 3) Pulses/legumes, g/day | No specific cut-off^b^ | 0 points: <20  1 point: 20-49  2 points: 50-79  **3 points: ≥80** |
| 4) Nuts and seeds, g/day | 20-30 | 0 points: 0  1 point: 1-9  2 points: 10-19  **3 points: 20-30**  2 points: 31-40  1 point: 41-50  0 point: >50 |
| 5) Fish and seafood, g/week | 300-450 | 0 points: <100  1 point: 100-199  2 points: 200-299  **3 points: 300-450**  2: points: 451-550  1 point: 551-650  0 points: >650 |
| 6) Red meat, g/week | ≤350 | 0 points: >500  1 point: 426-500  2 points: 351-425  **3 points: ≤350** |
| 7) Milk and dairy, g/day | 350-500 | 0 points: <150  1 point: 150-249  2 points: 250-349  **3 points: 350-500**  2 points: 501-599  1 point: 600-699  0 points: >700 |
| 8) Vegetable oils, g/day | ≥25 | 0 points: <5  1 point: 5-15  2 points: 16-24  3 points: ≥25 |
| 9) Sweets and confectioneries, incl. sugar-sweetened beverages, g/day | No specific cut-off^c^ | 0 points: >200  1 point: 150-199  2 points: 100-149  **3 points: <100** |
| 10) Alcohol, g/day | No safe limit | 0 points: >12  1 point: 7-12  2 points: 1-6  **3 points: 0** |
| ^a^Guidelines also include potatoes, white meat, and eggs, but as no specific cut-offs for intake have been set in the NNR for these, they were not included in our score.  ^b^Legumes/pulses are recommended as a significant part of the Nordic diet to support nutrient and protein intake, although it does not have a specified intake cut-off. Based on the Swedish Food Composition Database from the Swedish National Food Agency, a standard portion of legumes (beans and lentils) is approximately 75-100 grams per day [23]. Therefore, we defined the recommended intake as 80 grams of legumes per day, which was given 3 points in our score.  ^c^The guidelines for sweets, including chocolate, cakes, biscuits, confectioneries, and sugar-sweetened beverages, lacks a specified intake limit although their high energy and added sugar content warrants limited consumption [24]. The recommendation for added sugar is less than 10% of the total energy, corresponding to approximately 50-75 grams of free sugar per day for an adult [24]. Therefore, we defined the recommended level of sweets as less than 100 grams per day, which includes the total weight of foods containing added sugar. | | |

**
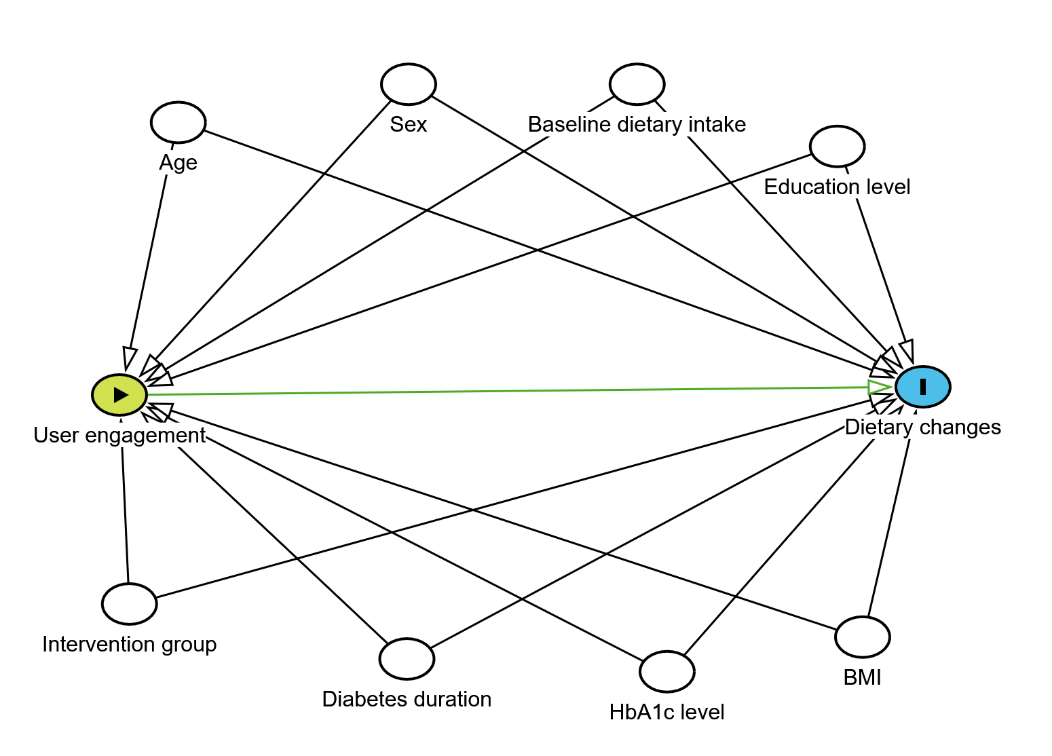
**

**Figure S1.** A Directed Acyclic Graph (DAG) displaying potential confounders in the association between user engagement and dietary changes.
